# Sympathetic nervous system activity in health and disease—advances with microneurographic recordings

**DOI:** 10.3389/fphys.2012.00470

**Published:** 2012-12-17

**Authors:** Elisabeth Lambert

**Affiliations:** ^1^Human Neurotransmitters Laboratory, Baker IDI Heart and Diabetes InstituteMelbourne, VIC, Australia; ^2^Department of Physiology, Monash UniversityMelbourne, VIC, Australia

The recent development and implementation of a catheter-based approach to denervate the renal sympathetic nerves in patients with resistant hypertension has ensured that the sympathetic nervous system (SNS) remains on center stage in cardiovascular medicine (Krum et al., 2009). Given this current interest in the field, the research topic “SNS activity in health and disease—advances with microneurographic recordings” is timely as it highlights the technique of microneurography, the only method available that directly assesses sympathetic nerve firing in the clinical setting. Microneurography permits the assessment of muscle (vasoconstrictor) sympathetic nerve activity (MSNA) in humans and was introduced over 40 years ago (Hagbarth and Vallbo, 1968). The eight articles compiled demonstrate how the technique has evolved and, importantly, how it has facilitated the understanding of sympathetic regulation in the healthy state as well as the sympathetic neural abnormalities underlying a number of conditions ranging from poor orthostatic control to chronic diseases linked with elevated cardiovascular risk.

Sympathetic neural drive is dependent on a range of factors including race, sex, and age. This idea is developed further in the review of Fu (2012) who points out that the sex and age-related differences in the prevalence of hypertension and cardiovascular disease in humans may be determined, at least in part, by differences in sympathetic control. Evidence derived from studies examining MSNA during phases of the menstrual cycle, pre- and post-menopause or during pregnancy highlights the role of sex hormones in neural control in women. Fu also draws attention to a number of conditions such as postural tachycardia, obesity, polycystic ovary syndrome, hypertensive pregnancy, essential hypertension, heart failure, and myocardial infarction where elevated sympathetic tone plays a major role in disease severity and prognosis. In line with the concept of age and sex differences in the control of SNS activity, the original contribution from Barnes et al. (2012) reveals distinct differences between young women, young men, and older women with regards to sympathetic and cardiac baroreflex sensitivity in response to a hypotensive challenge, again highlighting the potential importance of female sex hormones in sympathetic control. Multiunit MSNA has also been extensively used to investigate conditions linked with orthostatic intolerance. Ichinose and Nishiyasu (2012) and Ryan et al. (2012) extensively studied neural responses during syncope or until the point of decompensation induced by haemorrhage. Ichinose and Nishiyasu (2012) used multiunit MSNA to assess the arterial baroreflex (ABR) control under orthostatic stress and proposed that enhancement of the ABR control under orthostatic stress is an excellent defence against orthostatic hypotension and suggest that impairment of ABR control over sympathetic vasomotor activity leads to the severe hypotension associated with orthostatic syncope. Ryan et al. (2012) discuss the possible mechanisms involved in eliciting alterations in sympathetic activation to explain hemodynamic decompensation during severe hypovolemia. Altogether, these articles clearly indicate that multiunit MSNA provides valuable insight into the role of the SNS in the maintenance of blood pressure during various challenges. Nonetheless, the use of multiunit MSNA is not limited to this type of investigations and, as reviewed by Bruno et al. (2012), recent studies using multiunit MSNA recordings have highlighted that the SNS may be critically influenced, at the central and at the peripheral level, by factors regulating vascular function such as nitric oxide, reactive oxygen species, endothelin, and the renin-angiotensin system. This is an important issue and certainly merits further investigation as the interaction between the SNS and endothelial function may be of important clinical significance (Sverrisdottir et al., 2010).

Three articles acknowledge the limitations of multiunit MSNA and emphasize the increased information associated with recording single unit MSNA. Single unit recording is technically more difficult; however, it provides a more precise description of the way vasoconstrictor neurons behave during a sympathetic burst. Upon our previous findings, we (Lambert et al., 2012) highlight the fact that in conditions associated with anxiety disorders, single unit recording uncovers an atypical firing pattern of vasoconstrictor neurons even though multiunit MSNA is normal. We discuss the role of the firing pattern in sympathetic neural adaptation to long-term sympatho-inhibition seen during weight loss and the possible implication of aberration in the firing pattern in cardiovascular health taking into consideration that multiple firing during a sympathetic burst may carry adverse end organ consequences. Similarly, in their review, Murai et al. (2012) emphasize that in congestive heart failure (CHF) patients, the level of multiunit MSNA is nearly maximal at rest, so the frequency of pulse synchronized MSNA cannot increase further. Thus, single unit MSNA analysis is more useful for determining actual sympathetic neural firing within one cardiac interval. For example, they show that the firing of multiple spikes within one cardiac interval is significantly augmented during the Valsalva maneuver and exercise. This response is thought to influence strong effector organ responses, increasing peripheral vascular tone and may be seen as a contributor to exercise intolerance in these patients. This idea is reinforced by the findings that aberrant sympathetic firing pattern is associated with large noradrenaline release from the heart (Lambert et al., 2011). Macefield et al. (Macefield, 2012) reinforces the idea of the superiority of single-unit recordings compared to multiunit MSNA in conditions such as obstructive sleep apnea, chronic obstructive pulmonary disease, and bronchiectasis. These conditions are all associated with an elevation in multiunit MSNA to the same extent to that commonly seen in CHF, yet only single-unit MSNA reveals great differences in the sympathetic firing pattern in these respiratory diseases.

The SNS has long been recognized to play a central role in cardiovascular regulation, and targeting sympathetic activity is certainly a therapeutical approach in the treatment of many cardiovascular diseases. Thus, a reliable tool to assess SNS activity such as microneurography remains essential in order to comprehend the physiological mechanisms underlying disturbed SNS activity and to assess the effects of therapeutical strategies. It is anticipated that assessment of both multiunit and single unit will remain central in future investigations related to cardiovascular health.
